# Mutual regulation between CHD5 and EZH2 in hepatocellular carcinoma

**DOI:** 10.18632/oncotarget.5724

**Published:** 2015-10-26

**Authors:** Cheng-Rong Xie, Zhao Li, Hong-Guang Sun, Fu-Qiang Wang, Yu Sun, Wen-Xiu Zhao, Sheng Zhang, Wen-Xing Zhao, Xiao-Min Wang, Zhen-Yu Yin

**Affiliations:** ^1^ Department of Hepatobiliary Surgery, Zhongshan Hospital, Xiamen University, Fujian Provincial Key Laboratory of Chronic Liver Disease and Hepatocellular Carcinoma, Fujian, P.R. China

**Keywords:** EZH2, CHD5, hepatocellular carcinoma, prognosis, invasion

## Abstract

Chromodomain helicase DNA binding protein 5 (CHD5) acts as a tumor suppressor in many cancers. In the present study, we demonstrated that reduced levels of CHD5 in hepatocellular carcinoma (HCC) tissues were significantly associated with metastasis and poor prognosis. Gain-of-function assays revealed that CHD5 suppressed motility and invasion of HCC cells. Subsequent investigations showed that CHD5 was epigenetically silenced by polycomb repressive complex 2 (PRC2)-mediated the trimethylation of histone H3 at lysine 27 (H3K27me3) in HCC cells. Furthermore, overexpression of CHD5 repressed enhancer of zeste homolog 2 (EZH2) and activated PRC2 target genes, such as *p16* and *p21*. Chromatin immunoprecipitation and luciferase reporter assays also showed that CHD5 and EZH2 bind to each other's promoters and inhibit transcription. These findings uncovered, for the first time, a mutual suppression regulation between CHD5 and EZH2, which may provide new insights into their potential therapeutic significance for HCC.

## INTRODUCTION

Hepatocellular carcinoma (HCC) is the fifth most frequently occurring cancer worldwide [1]. Because of its high potential for metastasis and recurrence after surgical resection, prognosis of HCC patients remains very poor, despite advances in HCC treatments [2, 3]. Therefore, understanding the molecular mechanisms involved in carcinogenesis and recurrence, and identifying novel prognostic molecular biomarkers, will contribute to the development of effective therapeutic strategies for HCC.

There are several different classes of chromatin regulators, such as those that take part in “writing” and “reading” histone posttranslational modifications, which have been shown to be centrally involved in gene expression control during cancer occurrence and progression [4, 5]. For example, the polycomb group (PcG) proteins are well-characterized transcriptional repressors that regulate several developmental and physiological processes [6]. Enhancer of zeste homolog 2 (EZH2), a core component of the polycomb repressive complex 2 (PRC2), is a “writer” protein that catalyzes the trimethylation of histone H3 at lysine 27 (H3K27me3) and suppresses gene expression [7]. Previous studies showed that EZH2 overexpression is closely associated with the malignant progression and aggressive phenotypes of HCC [8–10]. Chromatin “reader” proteins control gene expression via reading and specifically binding to the N-terminus of post-translationally modified histones through conserved structural domains such as chromodomains, plant homeodomains (PHDs), and Tudor domains [11, 12]. The chromodomain helicase DNA-binding protein (CHD) family, which takes part in nucleosome remodeling and the regulation of gene expression, is structurally characterized by two N-terminal chromodomains and a helicase-like ATPase motif [13]. Several members of this family have been confirmed to play important roles in tumorigenesis and metastasis. CHD5 was recently found to be a potential tumor suppressor gene in cancer [14]. *CHD5* resides on the chromosomal locus 1p36 and has been reported to be silenced by genetic lesions [14], promoter DNA hypermethylation [15–17], histone demethylase JMJD2A, and micro-RNA 211 [18, 19] in many cancers. CHD5 inhibits proliferation and promotes apoptosis and senescence via the p19^Arf/^p53 pathway [14], in addition to the association with PHD-mediated histone 3 binding [20]. However, the suppressive function of CHD5, the mechanism of CHD5 inactivation, and the relationship with other “writer” proteins in HCC have not been well elucidated. In the present study, we showed that downregulation of CHD5 correlates with HCC metastasis and poor prognosis and that mutual suppression regulation occurs between EZH2 and CHD5 in HCC.

## RESULTS

### Underexpression of CHD5 is associated with HCC metastasis and poor prognosis

**Figure 1:**
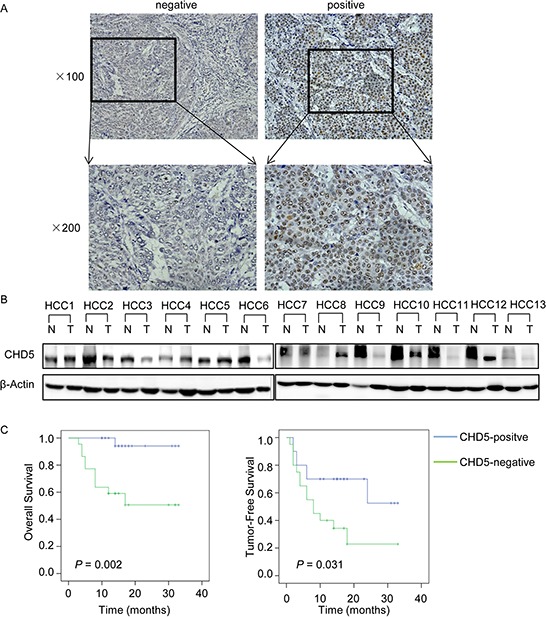
Expression of CHD5 in hepatocellular carcinoma (HCC) **A.** IHC analysis of CHD5 expression in 55 pairs of HCC tissues. **B.** Western blotting analysis of CHD5 expression in 13 representative HCC (T) tissues and adjacent non-cancerous (N) tissues. β-Actin was used as a loading control. **C.** Kaplan-Meier analysis of the correlation between CHD5 expression and overall survival or tumor-free survival of HCC patients.

To investigate the expression of *CHD5* in HCC patients, we measured CHD5 protein levels in 55 pairs of HCC and adjacent non-cancerous tissues by IHC and western blot analyses (Figure 1A and 1B). We detected positive signals in approximately half of the primary HCC samples (50.9%). CHD5 expression was much lower in 63.6% of HCC tissues compared with adjacent non-cancerous tissues. We further examined the correlation between CHD5 expression in primary HCC samples and clinicopathological characteristics of HCC patients. As shown in Table 1, statistical analyses indicate that CHD5 expression strongly correlates with HCC metastasis (*P* = 0.042) and recurrence (*P* = 0.022). Furthermore, Kaplan-Meier analyses revealed that underexpression of CHD5 significantly correlates with reduced overall survival and tumor-free survival rates (*P* = 0.002 and *P* = 0.031, respectively; Figure 1C). Taken together, these findings demonstrated that loss of CHD5 was associated with metastasis and poor prognosis in HCC.

**Table 1:** Correlation between CHD5 expression and clinicopathological characteristics of 55 HCCs

Parameters	CHD5		*P* values
	positive	negative	
Age			
<60	20	22	0.38
≥60	8	5	
Gender			
Male	23	22	1
Female	5	5	
AFP (ng/mL)			
<25	10	12	0.509
≥25	18	15	
Size (cm)			
<3	3	2	1
≥3	25	25	
Recurrence (36months)			
No	18	9	0.022*
Yes	10	18	
Liver cirhosis			
No	10	7	0.432
Yes	18	20	
Invasion			
No	11	4	0.042*
Yes	17	23	

AFP, alpha-fetoprotein

* *P* < 0.05

### Restoring expression of CHD5 decreases cell motility and invasion

**Figure 2:**
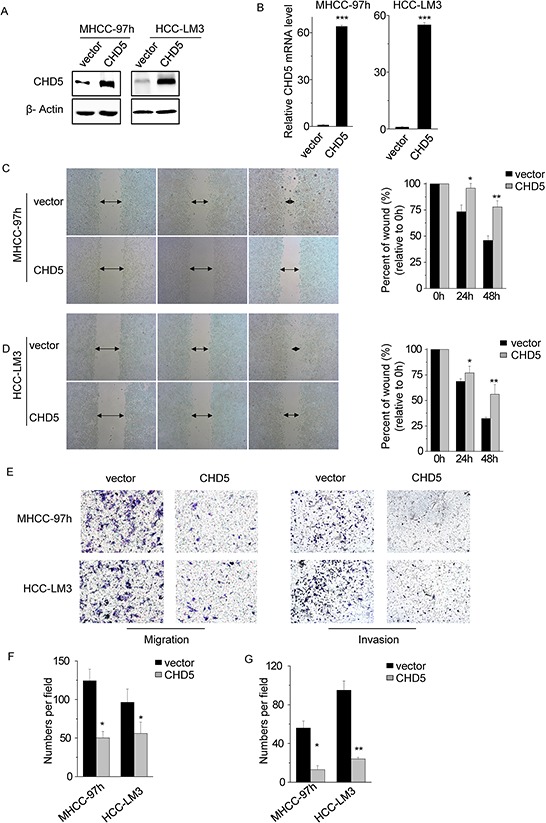
Restoring CHD5 expression decreases cell motility and invasion **A.** and **B.** The protein (A) and mRNA (B) levels of CHD5 were significantly increased following transfection with lentiviruses expressing CHD5 versus those observed following transfection with an empty vector in MHCC-97 h and HCC-LM3 cells. **C.** Significant impairment of wound-healing ability was found in MHCC-97 h cells expressing CHD5, compared to the control. **D.** Significant impairment of wound-healing ability was found in HCC-LM3 cells expressing CHD5, compared to the control. **E. F.** and **G.** The number of migrated (F) and invaded (G) cells in the CHD5-transfected group was decreased compared with that in the control. Data are shown as mean ± SD; *P < 0.05, ^**^P < 0.01, ^***^P < 0.001 (Student's *t* test).

A previous study demonstrated that restoration of CHD5 in HCC cell lines suppresses cell proliferation, colony formation, and tumorigenicity [14, 15]. Consistent with that previous study, we found that CHD5 overexpression inhibited cell proliferation and promoted apoptosis (Supplementary Figure S1). Our correlation analyses between CHD5 expression and clinicopathological features also suggested that CHD5 may decrease cell motility and invasion. To test this hypothesis, we overexpressed full-length CHD5 in MHCC-97 h and HCC-LM3 cells (Figure 2A and 2B) and performed migration, Matrigel invasion, and *in vitro* scratch wound healing assays. Wound healing (Figure 2C and 2D) and migration assays (Figure 2E and 2F) showed that ectopic expression of CHD5 decreased cell motility in both MHCC-97 h and HCC-LM3 (*P* < 0.05). Similarly, the Matrigel invasion assay (Figure 2E and 2G) showed that cells overexpressing CHD5 were significantly less invasive than control cells (*P* < 0.05). These observations indicate that CHD5 inhibits cell motility and invasion.

### PRC2 is involved in CHD5 down-regulation

**Figure 3:**
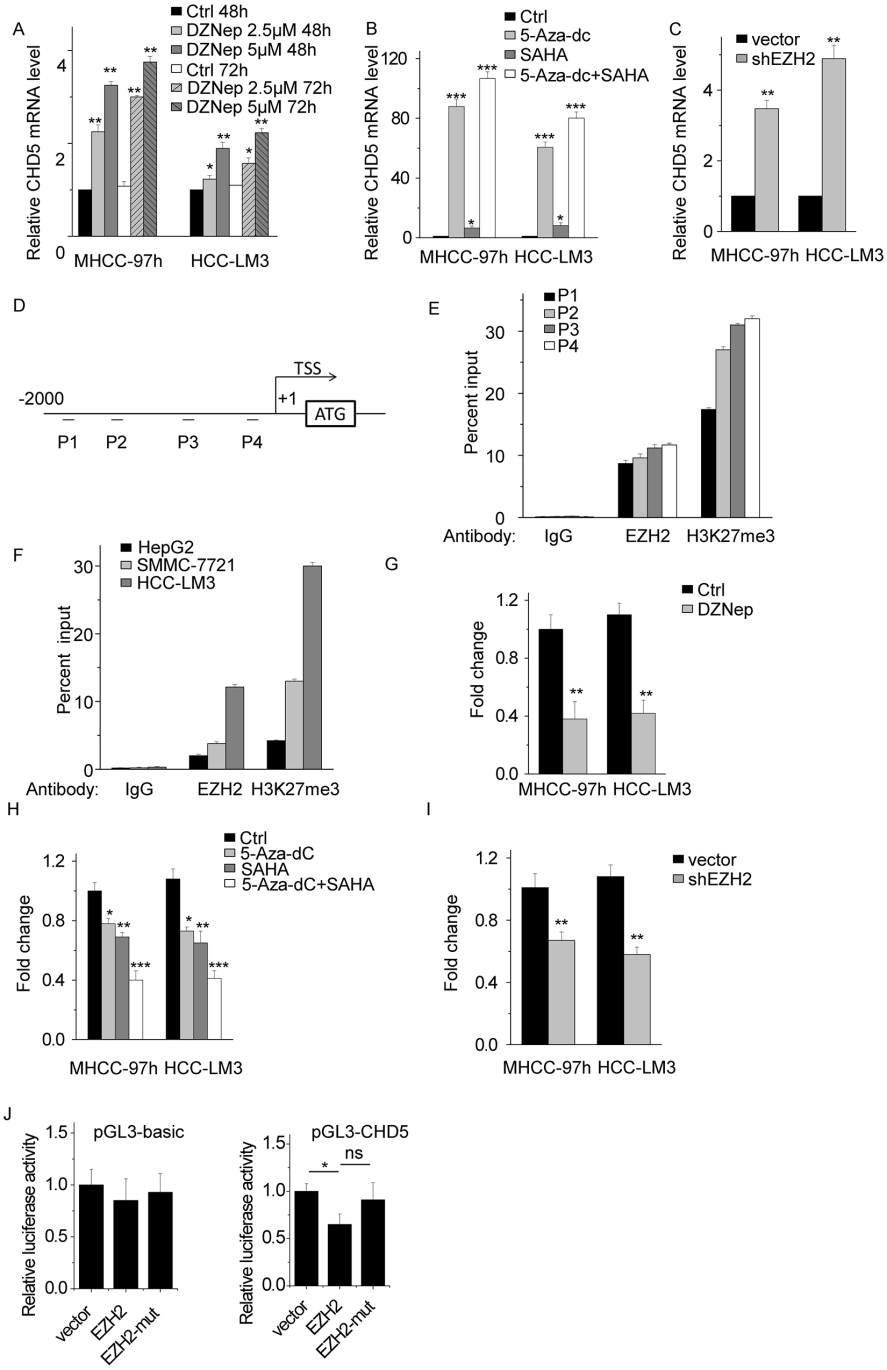
EZH2-mediated H3K27me3 is involved in CHD5 suppression **A.** Quantitative real-time PCR (qPCR) analysis of CHD5 expression in MHCC-97 h and HCC-LM3 cells by using DZNep at two different doses and time points. **B.** qPCR analysis of CHD5 expression in MHCC-97 h and HCC-LM3 cells by using 5-Aza-dc and/or SAHA. **C.** qPCR analysis of CHD5 expression in MHCC-97 h and HCC-LM3 cells in which EZH2 was knocked down by using shRNA. **D.** and **E.** Four sets of primers used for CHD5 promoter ChIP are shown. **F.** ChIP-qPCR analysis of EZH2 and H3K27me3 at *CHD5* promoter in HepG2, SMMC-7721, and HCC-LM3 cells. **G.** ChIP-qPCR analysis of H3K27me3 at *CHD5* promoter in MHCC-97 h and HCC-LM3 cells treated with DZNep. **H.** ChIP-qPCR analysis of H3K27me3 at *CHD5* promoter in MHCC-97 h and HCC-LM3 cells treated with 5-Aza-dc and/or SAHA. **I.** ChIP-qPCR analysis of H3K27me3 at *CHD5* promoter in MHCC-97 h and HCC-LM3 cells transfected with EZH2 shRNA. **J.** Luciferase activity of the empty vector and CHD5 promoter was measured after transfection of the EZH2 and EZH2 mutant with deletion of the SET domain. (pGL3-CHD5) : vector vs EZH2 is “*”; vector vs EZH2-mut is “ns”; EZH2 vs EZH2-mut is “*”. * Data are shown as the mean ± SD; *P < 0.05, **P < 0.01, ***P < 0.001 (Student's *t* test).

We have previously shown that the *CHD5* promoter is strongly methylated in HCC [21], which leads to a reduction in CHD5 expression. Other epigenetic mechanisms may contribute to CHD5 suppression. We investigated whether PRC2 is involved in this process. First, we treated MHCC-97 h and HCC-LM3 cells with deazaneplanocin A (DZNep), a global histone methylation inhibitor that depletes PRC2 and inhibits H3K27me3 [22]. DZNep treatment increased CHD5 expression in a concentration- and time-dependent manner (Figure 3A).

EZH2 also directly or indirectly facilitates DNA methylation and requires histone deacetylase (HDAC) to be functional [23, 24]. We predicted that chemical inhibitors of HDAC and other DNA methylation inhibitors would counteract PRC2-mediated CHD5 suppression. We treated MHCC-97 h and HCC-LM3 cells with the HDAC inhibitor suberoylanilide hydroxamic acid (SAHA) and/or the DNA methylation inhibitor 5-Aza-20-deoxycytidine (5-Aza-dc). As a result, CHD5 expression significantly increased upon treatment with SAHA, 5-aza-dc, or SAHA and 5-aza-dc (Figure 3B). We also transfected cells with EZH2 shRNA and measured CHD5 mRNA levels, and found that CHD5 expression levels were much higher in EZH2-shRNA-transfected cells (Figure 3C).

To confirm that EZH2 directly regulates *CHD5* by epigenetic suppression, we performed ChIP assays by using EZH2 and H3K27me3 antibodies in MHCC-97 h cells. We designed four sets of primers that bind different regions of the *CHD5* promoter (Figure 3D). As expected, EZH2 and H3K27me3 generally occupied the *CHD5* promoter region (Figure 3E). The primer set P4, which covers nucleotides from −369 to −161 base pairs, was tested and used in subsequent ChIP experiments. We also analyzed other HCC cell lines such as HepG2, SMMC-7721, and HCC-LM3 (Figure 3F). All of these cell lines had different levels of EZH2 and H3K27me3 occupancy at the *CHD5* promoter region, and the level of EZH2 and H3K27me3 occupancy was negatively correlated with CHD5 expression (Figure 3F and Supplementary Figure S2). In addition, we found that inhibition of EZH2 by shRNA or pharmacological inhibitors markedly decreased H3K27me3 occupancy at the *CHD5* promoter (Figure 3G, 3H, and 3I). For further confirmation, we constructed luciferase reporters containing 2000 nt of the *CHD5* promoter. We found that EZH2 reduced the luciferase activity of the CHD5 reporter vector but not that of the empty vector (Figure 3J). Overexpression of EZH2 with deletion of the SET domain (EZH2-mut) did not influence the luciferase activity of the CHD5 reporter vector. Taken together, these results indicate that *CHD5* is a direct target of PRC2, that EZH2 negatively regulates CHD5 expression via trimethylation of H3K27, and, possibly, that the SET domain of EZH2 is needed for EZH2-mediated CHD5 suppression.

### Expression of EZH2 and CHD5 is negatively correlated in HCC tissues

**Figure 4:**
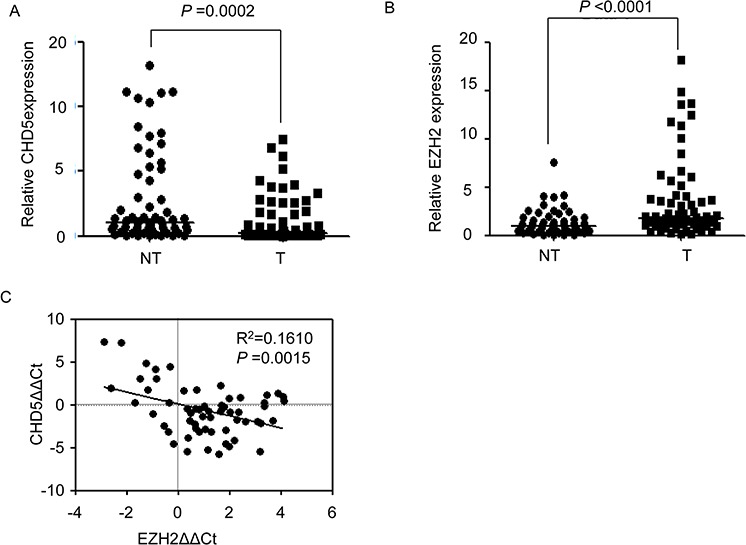
Correlation between CHD5 and EZH2 expression in HCC samples **A.** and **B.** qPCR analysis of CHD5 (A) and EZH2 (B) mRNA expression in 55 pairs of HCC (T) and adjacent non-cancerous tissues (NT). **C.** Correlation between CHD5 and EZH2 expression in 55 paired HCCs and matched non-cancerous tissues, with linear regression lines and Pearson correlation significance (Pearson Chi-square Test).

To further confirm the relationship between EZH2 and CHD5, we measured mRNA levels of EZH2 and CHD5 by quantitative real-time PCR (qPCR) in the 55 pairs of HCC and adjacent non-cancerous tissues. We found that CHD5 mRNA was underexpressed (fold change <0.5) in 54.5% of HCC tissue samples (Figure 4A), and EZH2 mRNA was overexpressed (fold change >2) in 58.2% of HCC tissues (Figure 4B). The fold change median for CHD5 expression in tumor tissues was significantly lower than that in adjacent non-cancerous tissues (0.227 versus 1; *P* = 0.0002, paired nonparametric test; Figure 4A), and the fold change median of EHZ2 expression in tumor tissues was significantly higher than that in adjacent non-cancerous tissues (2.225 versus 1; *P* < 0.0001, paired nonparametric test; Figure 4B). Finally, statistical analyses showed that CHD5 expression was negatively correlated with EZH2 expression in all 55 pairs of samples (R^2^ = 0.1610, *P* = 0.0015) (Figure 4C).

### *EHZ2* is directly targeted by CHD5

**Figure 5:**
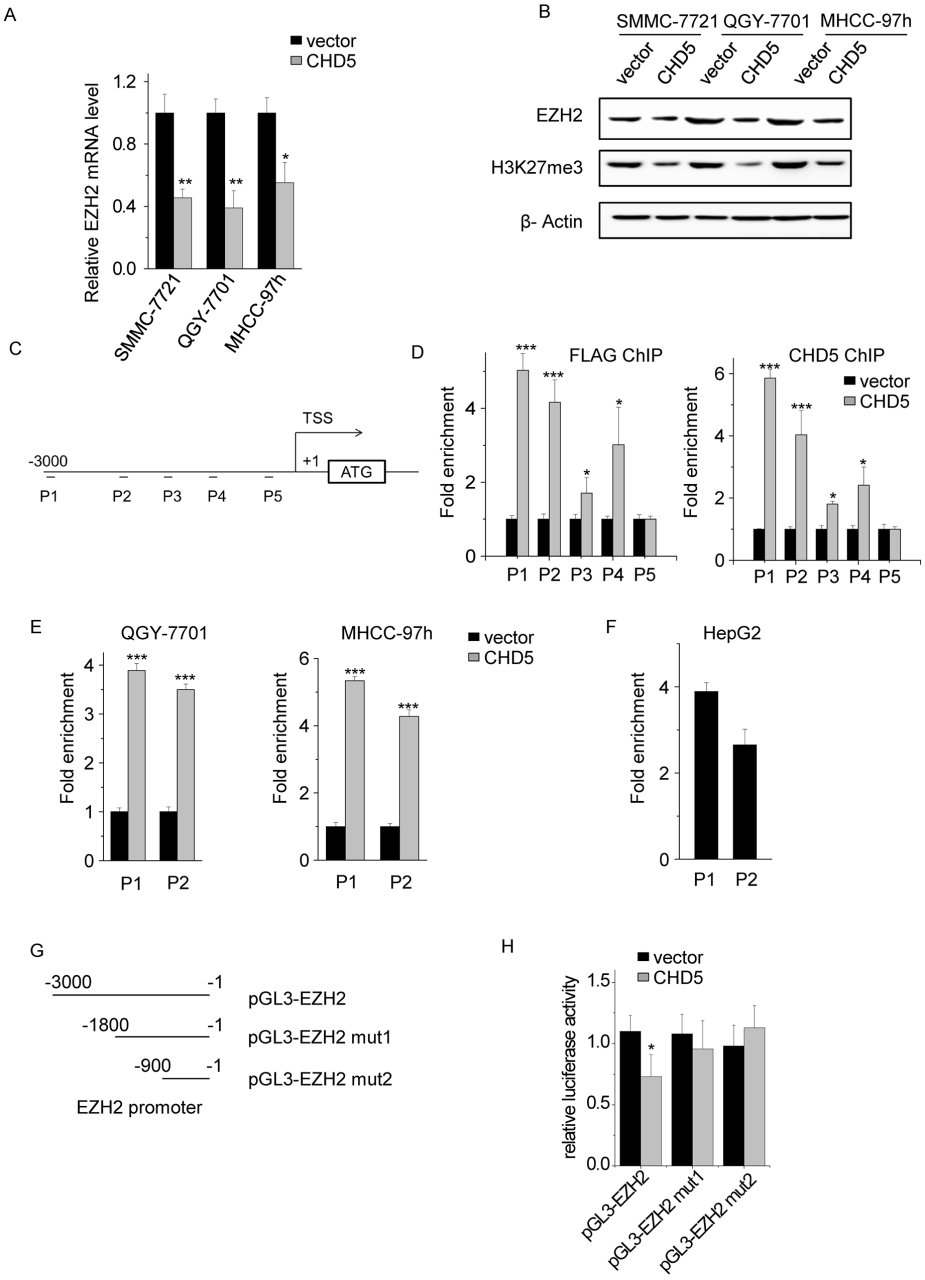
*EHZ2* is a direct target gene of CHD5 **A.** The mRNA levels of *EZH2* were quantitated by qPCR in CHD5-transfected QGY-7701, SMMC-7721, and MHCC-97 h cells. **B.** The protein levels of EZH2 and H3K27me3 were analyzed by western blotting in CHD5-transfected QGY-7701, SMMC-7721, and MHCC-97 h cells. **C.** Five sets of primers used for EZH2 promoter ChIP were shown. **D, E.** and **F.** ChIP assay demonstrated the binding of CHD5 to the EZH2 promoter. qPCR was performed to detect the amount of immunoprecipitated products. **G.** Deletion analysis of EZH2 promoter. **H.** Luciferase activity of the empty vector, EZH2 promoter, and EZH2 promoter mutants was measured after CHD5 transfection. Data are shown as mean ± SD; *P < 0.05, ^**^P < 0.01, ^***^P < 0.001 (Student's *t* test).

The negative correlation between CHD5 and EZH2 expression suggested that CHD5 may inhibit EZH2 expression. To test this hypothesis, we transfected MHCC-97 h, SMMC-7721, QGY-7701, and HCC-LM3 cells either with CHD5 or with an empty vector used as a control. We then measured EZH2 mRNA levels by qPCR. Ectopic CHD5 decreased EZH2 expression in MHCC-97 h, SMMC-7721, and QGY-7701 cells (Figure 5A) but not in HCC-LM3 cells (data not shown). In addition, we found decreased protein levels of EZH2 and H3K27me3 in CHD5-transfected MHCC-97 h, SMMC-7721, and QGY-7701 cells (Figure 5B).

To confirm that CHD5 directly suppresses EZH2 transcription, we transfected SMMC-7721 cells with FLAG-tagged CHD5, and performed ChIP assays by using CHD5 and FLAG antibodies. We designed five sets of primers binding different regions of the *EZH2* promoter (Figure 5C). We did not observe significant enrichment of promoter sequences in CHD5 pull-downs in control cells (Figure 5D). CHD5 and FLAG generally occupied the *EZH2* promoter region in CHD5-transfected cells (Figure 5D). Primer sets P1 and P2, covering nucleotides from −2810 to −2663 and from −2043 to −1952, respectively, were tested and used in subsequent ChIP experiments. We obtained similar results in QGY-7701 and MHCC-97 h cells (Figure 5E). We also tested whether endogenous CHD5 bound to the *EZH2* promoter. Enrichment of CHD5 in the *EZH2* promoter region in HepG2 cells that had high expression of CHD5 confirmed that endogenous CHD5 directly targets *EZH2* (Figure 5F and Supplementary Figure S2). To further determine whether CHD5 inhibits *EZH2* transcription, reporter constructs containing serial 5′ deletions of the *EZH2* promoter ([−3000/−1] EZH2, [−1800/−1] EZH2-mut1 and [−900/−1] EZH2-mut2) were cotransfected with CHD5 (Figure 5G). The luciferase reporter assay showed that CHD5 inhibited *EZH2* promoter activity and that the −3000 to −1800 region was essential for CHD5-mediated EZH2 suppression (Figure 5H). These results suggest that CHD5 inhibits EZH2 transcription in HCC cells.

### CHD5 activates EZH2 target genes through epigenetic mechanisms

**Figure 6:**
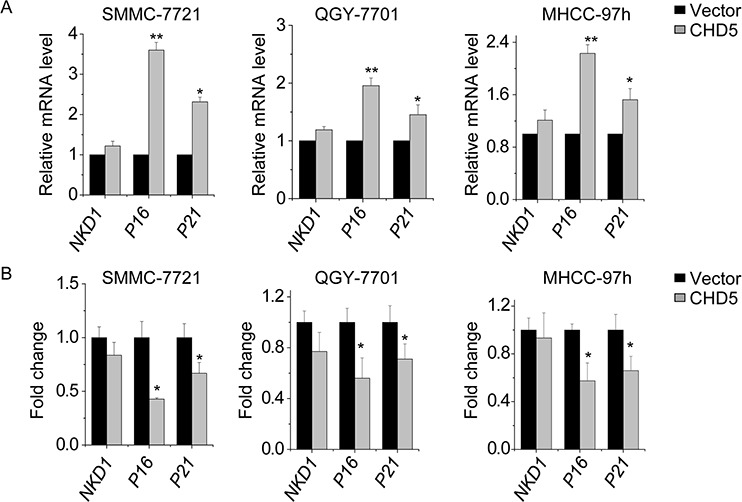
CHD5 activates EZH2 target genes through epigenetic mechanisms **A.** qPCR analysis of indicated genes in SMMC-7721, QGY-7701, and MHCC-97 h cells in which CHD5 was overexpressed by lentiviruses. **B.** ChIP-qPCR analysis of H3K27me3 in indicated genes in SMMC-7721, QGY-7701, and MHCC-97 h cells treated with CHD5-expressing lentiviruses. Data are shown as the mean ± SD; *P < 0.05, **P < 0.01 (Student's *t* test).

We investigated whether CHD5 stimulates expression the EZH2 target gene through epigenetic mechanisms. In particular, we measured mRNA levels of the EZH2 target genes *NKD1*, *p16*, and *p21* by qPCR in CHD5-transfected SMMC-7721, QGY-7701, and MHCC-97 h cells. CHD5 significantly increased p16 and p21 expression, but only slightly increased NKD1 expression (Figure 6A). We next quantified the H3K27me3 amount in the promoter regions of these three genes upon CHD5 overexpression. Levels of H3K27me3 were decreased in the promoter regions of *p16* and *p21*, but not in the *NKD1* promoter, as expected (Figure 6B).

## DISCUSSION

Deletions in the 1p36 region commonly occur in many types of malignant tumors, such as neural-related malignancies [25], hematopoietic malignancies [26], thyroid cancer [27], colon cancer [28], cervix cancer [29], and breast cancer [30]. Consequently, tumor suppressor genes located in this region, such as *CHD5*, are either lost or inactivated in these cancers [14]. In the present study, we found that both mRNA and protein levels of CHD5 were decreased in HCC tissues relative to those in non-cancerous tissues. Although deletions on 8p, 13q, and 17p are common in liver cancer, chromosomal aberration of 1p36 is rare in HCC [31, 32]. Therefore, down-regulation of CHD5 in HCC is more likely due to epigenetic mechanisms. Recent studies have demonstrated that CHD5 expression is epigenetically silenced by promoter DNA hypermethylation in HCC [15], colorectal cancer [33], breast cancer [34], gastric cancer [17], and lung cancer [35]. Accumulating evidence suggests that increased activity of EZH2 is oncogenic as measured by cell proliferation, cell invasion, and tumorigenesis [36–38]. Overexpression of EZH2 has been shown to promote cell proliferation by repressing the cell-cycle regulation genes *p16* and *p21* [39, 40], and to increase cell motility and invasion by inhibiting the metastasis-associated genes *CDH1* and *DLC1* [24, 41]. In addition, EZH2 also inhibits malignant phenotypes of HCC cells through suppression of miRNA, such as miR-200a/b and let-7c [42, 43]. We hypothesized that EZH2 may take part in CHD5 suppression. In this study, for the first time, we found that enrichment of H3K27me3 at the *CHD5* promoter region causes CHD5 epigenetic repression. Upon EZH2 silencing (with shRNA) or inhibition (with DZNep, 5-Aza-dc, or SAHA), CHD5 expression was restored and the H3K27me3 level at the *CHD5* promoter region was decreased. ChIP and luciferase reporter assays also showed that EZH2 directly binds to the *CHD5* promoter and inhibits its transcription activity. Thus, our study revealed that *CHD5* is a novel direct target of EZH2, and may be part of a tumor suppressor network that is suppressed by EZH2.

Emerging evidence suggests that silencing of CHD5 may contribute to carcinogenesis. CHD5 expression is associated with tumor grade and poor survival in primary gallbladder carcinoma patients [44]. However, the clinical significance of CHD5 in HCC is still unclear. To the best of our knowledge, this is the first study to report that loss of CHD5 expression significantly correlates with unfavorable clinicopathological features of HCC patients, including tumor metastasis, recurrence, poor overall and tumor-free survival. Furthermore, functional assays also showed that restoration of CHD5 inhibits cell motility and invasion. The negative correlation between CHD5 and EZH2 expression of HCC samples suggests that CHD5 may inhibit EZH2 expression. Further investigation found that CHD5 directly binds to the *EZH2* promoter and inhibits its transcription. CHD5 could directly bind to H3K27me3 [45], or interact with the nucleosome remodeling and deacetylase (NuRD) complex to repress target genes [46]. We suspected that CHD5 may recruit the NuRD complex and inhibit *EZH2* transcription. Because CHD5 decreases EZH2 expression and global H3K27me3, it is not likely to bind the decreased H3K27me3 of *EZH2* promoter to suppress its transcription. The mechanism by which CHD5 inhibits EZH2 expression requires further investigations. Although CHD5 has been shown to repress another PcG protein in neuroblastoma, BMI1 (a core component of PRC1) [20], we did not observe an influence of CHD5 on BMI1 expression in HCC cells (data not shown). CHD5 might suppress PcG protein expression in a tissue-specific manner.

In this study, we showed that EZH2 could transcriptionally repress *CHD5* expression. Additionally, ectopic expression of CHD5 reduced EZH2 expression. These data suggest the existence of a mutual suppression regulation between EZH2 and CHD5. Recent studies have reported a similar pattern in regulatory networks that play critical roles in human cancers [47–49]. In one study, BMI1 activated the WNT pathway by repressing the DKK family. Suppression of DKK1 up-regulated c-Myc, which in turn activated the transcription of BMI1 [48]. Another study reported that PRC2 represses several microRNAs, which in turn activates PRC1 and PRC2 expression [49]. These studies, along with ours, strongly suggest that dysregulation of feedback networks may contribute to cancer progression.

Pharmacologic targeting of dysregulated epigenetic modifications has emerged as an attractive approach in cancer therapy. DZNep, 5-Aza-dc, and SAHA have been shown to inhibit tumorigenesis and tumor progression *in vivo* [50–52]. Our findings suggest that these drugs inhibit PRC2 function and restore CHD5 expression. The therapeutic potential of DZNep, 5-Aza-dc, and SAHA against HCC development and recurrence still needs to be investigated.

In conclusion, we showed that CHD5 is epigenetically silenced by PRC2-mediated H3K27me3. Downregulation of CHD5 correlates with HCC metastasis and poor prognosis. Our findings also uncovered a mutual suppression regulation between CHD5 and EZH2.

## MATERIALS AND METHODS

### Patients and tissue samples

Fifty-five pairs of HCC tissues and adjacent non-cancerous tissues were collected from patients who initially underwent hepatectomy and were diagnosed with HCC between January 2011 and September 2013 at Zhongshan Hospital, Xiamen University. All subjects provided written informed consent. Samples were selected randomly. No patients were treated with chemotherapy and radiotherapy before hepatectomy. This study was approved by the ethics committee of Xiamen Zhongshan Hospital.

### Cell culture

HepG2, SMMC-7721, and QGY-7701 HCC cell lines were obtained from the Cell Bank of Type Culture Collection (Chinese Academy of Sciences). MHCC-97H and HCC-LM3 cells were obtained from Fudan University. Cell lines were maintained at 37°C, in an incubator with 5% CO_2_, in Dulbecco's Modified Eagle's Medium (HyClone) supplemented with 10% fetal bovine serum (Biological Industry).

### Plasmid construction, lentiviral construction, and cell transfection

Flag-tagged full-length human CHD5 cDNA was cloned into the lentiviral vector pBobi-CMV. 293T (ATCC) cells were transfected with lentiviral and packaging vectors by using TurboFect Transfection Reagent (Thermo). The medium was changed 24 h after transfection and the medium containing the lentivirus was collected 48 h later. Cells were infected with lentivirus in the presence of 10 μg/mL polybrene (Sigma).

### Western blot analysis

Cells and tissues were lysed in RIPA lysis buffer containing the protease inhibitor PMSF. Protein lysates were subjected to SDS-PAGE and transferred to PVDF membranes (Millipore). Membranes were incubated overnight with antibodies against CHD5 (Abcam, 1:1000) and β-actin (Santa Cruz Biotechnology, 1:1000) at 4°C. Secondary antibodies were goat anti-mouse-HRP and goat anti-rabbit-HRP (Pierce, 1:5000). After incubation with a secondary antibody, the membranes were exposed to ECL solution (Thermo). Experiments were performed at least three times independently.

### RNA isolation and quantitative real-time PCR analyses

Total RNA was extracted using TRIzol reagent (Invitrogen) and reverse-transcribed using PrimeScript RT Reagent Kit with gDNA Eraser (Takara). qPCR was performed using an ABI 7500 Real-Time PCR system and SYBR Green reagents (Takara). ACTB was used as an internal control. All experiments were performed in duplicate and repeated three times. Primers for qPCR are shown in Supplementary Table S1.

### Immunohistochemical staining

Immunohistochemical (IHC) staining was performed on formalin-fixed, paraffin-embedded (FFPE) tissues by using an IHC kit (Maixin). In brief, sections were deparaffinized and rehydrated. After blocking of endogenous peroxidase, antigen retrieval, and blocking of nonspecific binding proteins, the slides were incubated overnight with a 1:50 dilution of rat-monoclonal antibody against CHD5 (Millipore) at 4°C in a moist chamber. The specificity of this antibody has been stated and verified previously [53]. The slides were sequentially incubated with biotinylated goat anti-rat secondary antibody and then streptavidin-peroxidase conjugate, each for 30 min at room temperature. Finally, 3, 5-diaminobenzidine (DAB) was used for color development followed by hematoxylin counterstaining. CHD5 expression in tumor tissues detectable by IHC was defined as “positive,” whereas lack of detection was defined as “negative.”

### Chromatin immunoprecipitation assay

Chromatin immunoprecipitation (ChIP) was performed using an EZ-Magna ChIP A/G kit (Millipore) according to the manufacturer's instructions. Briefly, cells were cross-linked with 1% formaldehyde for 10 min at room temperature. Cross-linked chromatin was fragmented by sonication to an average size of 100–1000 base pairs. EZH2 antibodies (Millipore), H3K27me3 antibodies (Abcam), CHD5 antibodies (Santa Cruz), or IgG antibodies (Millipore) were mixed with nuclear lysates for immunoprecipitation. Co-precipitated DNA was purified, and the level of target genes was quantified using qPCR. Primer sequences are shown in Supplementary Table S1.

### Wound healing, cell migration, and invasion assays

Wound healing was assessed by measuring the movement of cells into a scraped, acellular area created by a 200-μL pipette tube. Cell spreading and wound closure were observed and photographed after 24 h and 48 h with a microscope. Cell migration and invasion assays were performed using transwell chambers with or without Matrigel (8 μm, Corning), according to the manufacturer's instructions. The number of cells that migrated and invaded through the membrane was counted in 10 fields with a 10× objective lens.

### Luciferase reporter assays

Luciferase activities were detected with the Dual Luciferase Assay Kit (Promega) according to the manufacturer's instructions.

### Statistical analyses

All statistical analyses were performed using SPSS software. The correlation between CHD5 expression and clinicopathological features was analyzed by a chi-squared test. Survival curves were calculated by Kaplan-Meier and log-rank test. *P* < 0.05 was considered statistically significant unless otherwise indicated.

## SUPPLEMENTARY MATEREALS AND METHODS FIGURES AND TABLE


