# Supplementary material for: Mutual regulation between CHD5 and EZH2 in hepatocellular carcinoma
Source: Oncotarget. 2015 Oct 26;6(38):40940–52. doi: 10.18632/oncotarget.5724 (PMC4747380; doi:10.18632/oncotarget.5724)
Supplement: Supplementary file 1 [file oncotarget-06-40940-s001.pdf]

SUPPLEMENTARY MATEREALS AND METHODS

MTT assays

HCC cells were seeded at a density of 1 × 10^3 cells per well in 96-well plates, the viability of the cells was assessed from three replicates in three independent experiments by the MTT (3–2,5-diphenyl tetrazolium bromide) assay (Sigma, St, Louis, MO).

Flow cytometry

Cells (1 × 10^6) were trypsinized and resuspended to generate single-cell suspensions.

To study apoptosis, cells were stained with fluorescein isothiocyanate-conjugated Annexin V and 7-AAD (Apoptosis Detection Kit, KeyGEN), as suggested by the manufacturer. Cells were then analyzed with a FACScan flow cytometer, and the data were studied using FlowJo software (Tree Star lnc).

SUPPLEMENTARY FIGURES AND TABLE

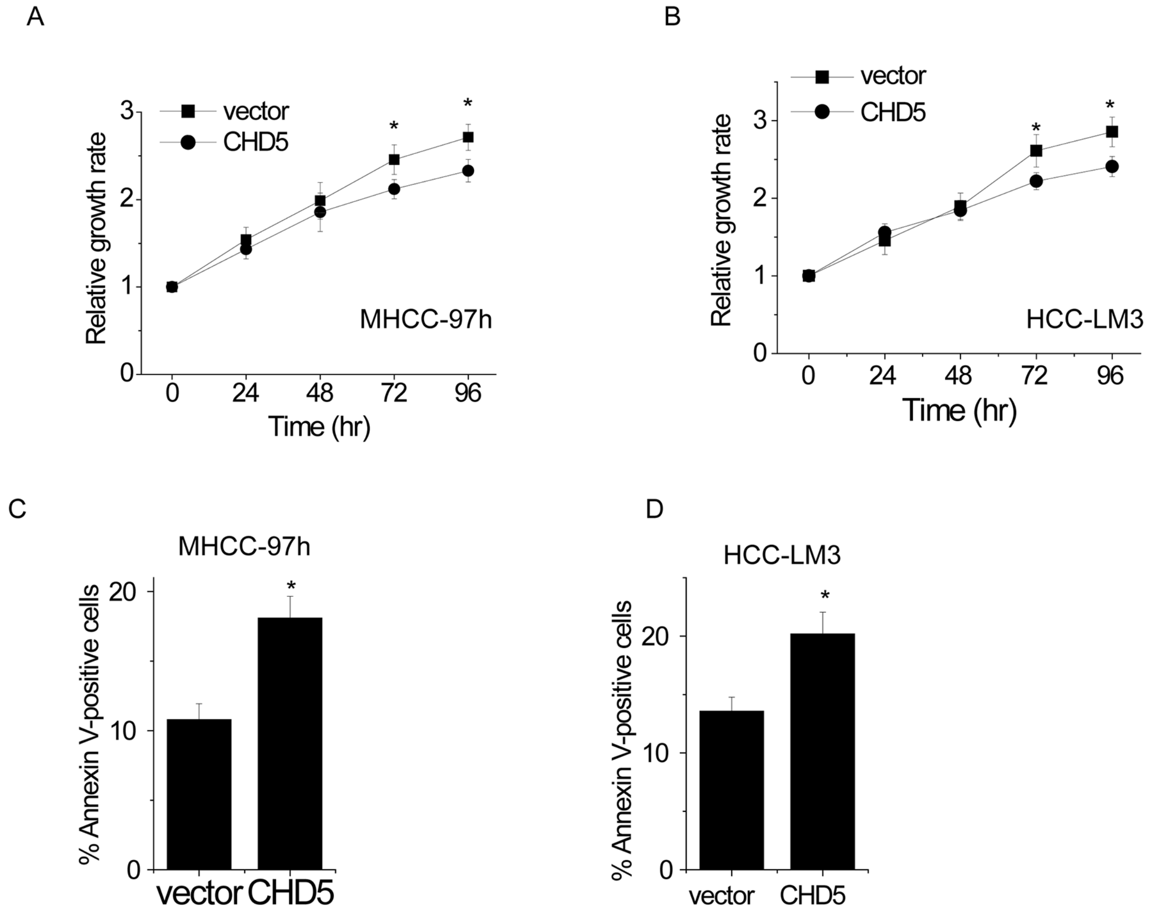

Supplementary Figure S1: Overexpression of CHD5 inhibited cell proliferation and promoted apoptosis. A. and **B.** CHD5 overexpression inhibited the proliferation of MHCC-97H (A) and HCC-LM3 (B) cells. **C.** and **D.** MHCC-97H (C) and HCC-LM3 (D) cells with CHD5 overexpression were stained with a combination of annexin V and 7-AAD and analyzed by FACS. Cells positive for annexin V staining were counted as apoptotic cells, and the percentage of apoptotic cells is shown. Data are shown as mean ± SD; $^{*}P < 0.05$, $^{**}P < 0.01$, $^{***}P < 0.001$ (Student's *t* test).

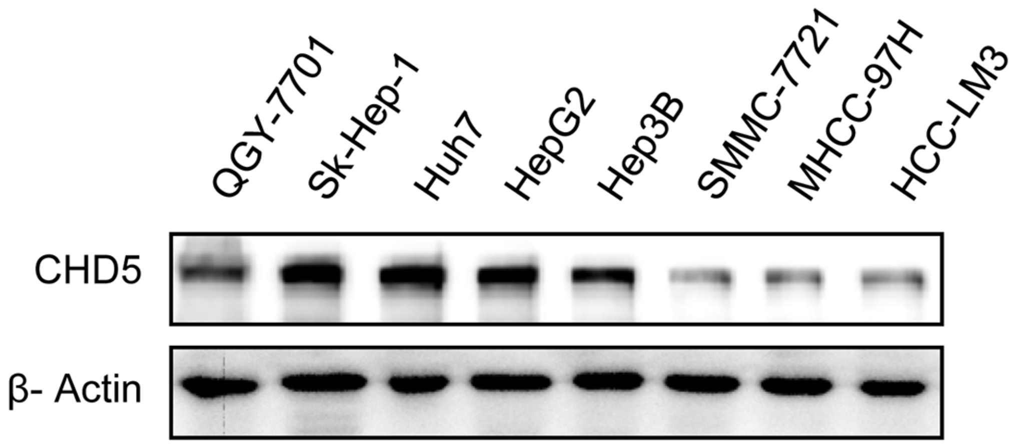

Supplementary Figure S2: CHD5 expression in different HCC cell lines.

Supplementary Table S1: Sequence for PCR

**Real-time PCR**		**sequence**
GAPDH	F	CTTTGGTATCGTGGAAGGACTC
	R	AGTAGAGGCAGGGATGATGT
CHD5	F	CACGGTGATGTATCGCAACTA
	R	GGTCCTTGTTCTTCCTCTTCTC
EZH2	F	GCAGAAAGATCTGGAGGATCAC
	R	TTCTTCTGCTGTGCCCTTATC
NKD1	F	CAGCGGAGATGAGAAGAAGATG
	R	CAAAGTCATACAGGGTGAAGGT
P16	F	GCCCAACGCACCGAATAGTTA
	R	ACGGGTCGGGTGAGAGT
P21	F	GTCACTGTCTTGTACCCTTGTG
	R	GGCGTTTGGAGTGGTAGAAA
**ChIP-qPCR**		
CHD5	1F	GCCTGTAATCCCAGCACTTT
	1R	CCACCACACCTGGCTAATTT
	2F	GGAATTGGAGCCAGGGTTAAT
	2R	CCAACACCTCTCTAATCCCTTTC
	3F	GGCTTTGGCCTCCATCTT
	3R	AAACTCCCGCTCAGAAGC
	4F	CCCAACTTGTTCTCTGCTGTTA
	4R	GCACACATGCCCGGATT

(*Continued*)

Real-time PCR		sequence
EZH2	1F	GTGCCACCACACTCAGTTAT
	1R	CAAGATATTCCAGGCCAGACTT
	2F	GGATCACAAAGTCAGGAGATCG
	2R	CCACCACACCCGACTAATTT
	3F	CCTCTAATTCCTCTGGGTTGAG
	3R	CTTGCAGTGAGCCGAGAT
	4F	CACAGGTTTCTAGGGCGATAAG
	4R	TCCCAAAGTGCTGGGATTAC
	5F	GTTGCGGTGAGTCGAGAT
	5R	CCGGACCCGTTACTACTTT
NKD1	F	TGCCAGGACGAGCGTAACA
	R	TGGTGGCGGTCGACATC
P16	F	AGGGGAAGGAGAGAGCAGTC
	R	GGGTGTTTGGTGTCATAGGG
P21	F	GCTTCAAGGCAGTGGGAGA
	R	CCAGGATTGTGGCTAAACC
